# Recent Advances in Nanoparticle-Mediated Diagnosis and the Treatment of Pancreatic Cancer

**DOI:** 10.3390/ijms22158060

**Published:** 2021-07-28

**Authors:** Andreea Nedelcu, Teodora Mocan, Cristiana Grapa, Lucian Mocan

**Affiliations:** 1 3rd Surgery Clinic, “Iuliu Hatieganu” University of Medicine and Pharmacy, 400158 Cluj-Napoca, Romania; a.popanedelcu@reginamaria.ro (A.N.); lucian.mocan@umfcluj.ro (L.M.); 2 Nanomedicine Department, Regional Institute of Gastroenterology and Hepatology, Octavian Fodor, 400158 Cluj-Napoca, Romania; GRAPA.CRISTIANA.MARIA@umfcluj.ro; 3 Regina Maria Hospital, 400158 Cluj-Napoca, Romania; 4 Physiology Department, “Iuliu Hatieganu” University of Medicine and Pharmacy, 400158 Cluj-Napoca, Romania

**Keywords:** pancreatic cancer, drug delivery, nanoparticles

## Abstract

Pancreatic cancer (PC), one of the most lethal solid tumors in humans, has a five-year survival rate of only 4%. Surgical treatment is the only accepted therapy with curative intent because the vast majority of these tumors are chemoresistant. Unfortunately, due to the aggressive nature of these tumors, fewer than 20% are resectable when the first symptoms occur. Novel therapies are required to overcome all these therapeutic issues, and the development of active nanocarriers represents an exciting opportunity to improve PC outcomes. The present review focuses on recent advances in the field of nanotechnology with application in PC treatment.

## 1. Introduction

Since nanomedicine-based approaches have been developed, physicians have used such techniques in the prophylaxis, detection and therapy of deadly illnesses, including cancer. The primary implementation of nanotechnology in specified drug delivery has been demonstrated by nanoparticles that are notably effective in the therapy of cancer.

Various elements of the living cells are genuinely in nanoscale sequence; therefore, it was expected that nanotechnology may be a worthwhile subject for study in biology and medicine. As an important component of nanotechnology domain, nanomedicine is a cross-disciplinary area that connects biology, chemistry, engineering and medicine to offer further effective instruments for the prophylaxis and therapy of multiple illnesses. 

Pancreatic ductal adenocarcinoma (PDAC) is the most lethal solid tumor in humans, with an average patient survival of 6–8 months post-detection (five-year survival 1–5%). PDAC prevalence is increasing, and it is anticipated to be the secondary source of cancer-related deaths by 2030.

The low survival incidence is a result of its extremely aggressive nature, in-built resilience to chemotherapeutics, and deficit of successful therapies, along with slow diagnosis as a consequence of unspecific manifestations and lack of quick diagnostic strategies. 

Even though, concurrently, the endocrine and the exocrine cells of the pancreas might convert to precancerous cells, pancreatic cancer (PaCa) build up from exocrine cells is far more frequent, and almost all PDAC tumors are adenocarcinomas. An important percentage of patients (~85%) are submitted with in situ progressed or unresectable disease whose evolution is practically uncontrollable with ongoing treatments; they unfortunately provide an insignificant effect on survival [1]. 

However, many of these problems have been fixed by identifying nanotechnology-based carrier methods that have been employed in imaging and therapy approaches as well. The major concern remains establishing more efficient detection methods and techniques in order to stop patients from being belatedly diagnosed and treated.

Further defiance in pancreatic cancer management is represented by the drug resistance challenge that can be cleared up by providing an in-depth insight of the microenvironment involved with the disease, in order to create ingenious nanocarriers. 

Although various impediments have affected advancements in developing efficient treatments, it has been discovered that one of the major aspects hampering the infiltration of chemotherapy medication is a lack of vascularization in combination with a heavy tumor stroma. Nanotechnology supplies methods to challenge the treatment-defiant status in cancer, via intelligent techniques of delivery for appropriate medication buildup in the tumor locations.

An upgraded penetration and withholding effect (Enhanced permeability and Retention, EPR) represent the foundation of the recently developed anticancer nanocarriers. EPR delivery techniques are based on the long-term observation that vascular architecture facilitates the strong inclusion of medication among the cancer environment [2].

In the present review, we demonstrate our main findings regarding advances in nano-mediated pancreatic cancer therapies and their potential use for clinical implementation. We included predominantly original articles, English-written, published from 2016 to 2020; other types of articles such as narrative reviews were included if they were related to our main topic. Our aim is to provide the reader with a clear overview on the role of nanotechnology in pancreatic cancer treatment and its main recent developments.

## 2. Hyaluronic Acid 

Hyaluronic acid-obtained nanoparticles bound to indocyanine green, named NanoICG, were used for the intraoperative assessment of pancreatic carcinoma extensions. NanoICG proved to be biocompatible and non-toxic following in vitro viability tests. Following its administration, it was observed mostly in the pancreas in an orthotopic pancreatic ductal adenocarcinoma model, and showed selective accumulation for pancreatic cancer cells, compared to non-malignant pancreatic tissue. Fluorescence microscopy also showed greater selectivity in pancreatic cancer tissue and splenic metastases of NanoICG in comparison to ICG alone.

The in vivo toxicity and biocompatibility tests on NanoICG-treated healthy mice showed insignificant systemic side effects. The presented results indicate that NanoICG is an encouraging contrast agent for the intraoperative recognition of malignant tumors [3].

## 3. Hyaluronic Acid and Gold Np

Establishing a prompt diagnosis and starting adequate treatment is crucial to maximize survival for patients with pancreatic cancer. Nevertheless, current imaging does not substantially amplify our ability to identify the disease in its early phases.

In one of their reports, Qiu et al. composed a multimodal nanosystem for the specific identification and efficient therapy of pancreatic cancer, based on gold nanocages (AuNCs) functionalized with hyaluronic acid (HA) and conjugated with anti-Glypican-1 (anti-GPC1) antibody, oridonin (ORI), gadolinium (Gd), and Cy7 dye. The authors estimated the features of GPC1-Gd-ORI@HAuNCs-Cy7 NPs (ORI-GPC1-NPs), as well as characteristics such as shape and surface modifications. Qiu et al. calculated the drug charging and distribution effectiveness using cell cultures. Near-infrared fluorescence (NIRF)/magnetic resonance imaging (MRI) and theranostic potential were quantified in vitro and in vivo. Overall, they discovered that ORI-GPC1-NPs exhibited specific internalization and fluorescent/MRI affinity. Bio-transmission electron microscopy (bio-TEM) analysis illustrated that ORI-GPC1-NPs were selectively endocytosed into PANC-1 and BXPC-3 (overexpression GPC1). No internalization occurred in 293 T cells (GPC1-negative). 

In comparison with ORI and ORI-NPs, ORI-GPC1-NPs substantially repressed the growth and increased the apoptosis in PANC-1 cell culture. In addition, in vivo toxicity tests indicated that ORI-GPC1-NPs had high biocompatibility. In vivo examinations showed that ORI-GPC1-NPs empowered multimodal imaging and specific therapy in pancreatic tumor xenografted mice.

In conclusion, Qiu et al. demonstrated that ORI-GPC1-NP can represent a powerful theranostic system for the specific imaging and efficient treatment of pancreatic carcinoma [4].

## 4. Hyaluronic Acid and Gemcitabine Np

One major health problem in oncology, that of chemotherapy resistance, can be overcome using a combination of drugs that act in various stages of the malignant cell development cycle. For instance, combining gemcitabine (GMC) with quercetin (QCT) demonstrated a considerable impact in suppressing the evolution of pancreatic cancer cells. 

GMC and QCT have been functionalized with biodegradable nanoparticles (NPs) based on poly (lactic-co-glycolic acid), adorned on the outside with hyaluronic acid (HA; viz., PPHA NPs), which display a high affinity for the CD44 receptor, and are upregulated in malign pancreatic tumors. 

The prepared HA-decorated NPs charged with GMC and QCT expressed low cytotoxic effect and selective internalization in two malignant pancreatic cell lines, Mia-PaCa-2 and PANC-1, in comparison with both chemotherapies alone and NPs-GMC-QTC without HA. HA-decorated NPs were showed to reduce the interleukin cellular levels in both cell lines, thus enabling a strong anti-inflammatory effect. 

This outcome is of great importance, considering the decisive role of interleukins in the development, extension, and chemoresistance of human pancreas cancer cells [5].

## 5. GOLD Np (+ Gemcitabine, + Varlitinib)

Standard oncologic treatment of pancreatic cancer (PaCa) have poor drug penetrability and increased chemoresistance. The development of new techniques for improved treatments is still problematic at present. 

Hereby, Lin et al. describe a novel ultrasound-targeted microbubble destruction (UTMD)-promoted distribution approach relying on dendrimer-entrapped gold nanoparticles (Au DENPs) for the co-delivery of gemcitabine (Gem) and miR-21 inhibitor (miR-21i). In this study, Gem-Au DENPs/miR-21i were developed and the polyplexes were characterized through transmission electron microscopy (TEM), gel retardation tests and dynamic light scattering (DLS). The cytotoxic and therapeutic effects of Gem-Au DENPs/miR-21i were examined in vivo by contrast-enhanced ultrasound (CEUS), hematoxylin and eosin (H&E) staining, TUNEL staining and comparisons of tumor volumes. The study showed that the Gem-Au DENPs/miR-21i can be selectively internalized inside pancreatic cancer cells by cancer cells; cellular intake was improved using an ultrasound-targeted microbubble destruction (UTMD) device (0.4 W/cm^2^) to enhance cellular internalization. Furthermore, the co-administration of Gem and miR-21i +/− UTMD induced 82-fold and 13-fold reduced half maximal inhibitory concentration (IC50) values compared with free Gem, respectively. The in vivo testing showed that following UTMD application, the co-distribution of Gem and miR-21i induced a significant decrease in tumor volume and enhanced arterial distribution in xenografted pancreatic tumors. 

The authors concluded that the therapeutic potential of Gem and miR-21i utilizing Au DENPs can be significantly improved using the UTMD technique; therefore, this offers an encouraging procedure for successful pancreatic cancer therapies [6].

## 6. Gold Np and Varlitinib

Pegylated gold nanoparticles were bioconjugated with varlitinib via a carbodiimide-mediated cross-linking mechanism and characterized using infrared and X-ray photoelectron spectroscopy. In vitro experiments on MIA PaCa-2 cells confirmed that PEGAuNPsVarl conjugates increased the varlitinib chemotherapeutic effect at very low absorption (IC50 = 80 nM) in comparison with varlitinib only (IC50 = 259 nM), and exhibited a full drug assimilation 72 hours after administration [7].

## 7. Gemcitabine (and Iron Oxide; and Simvastatin; and Relaxin)

**Figure 1:**
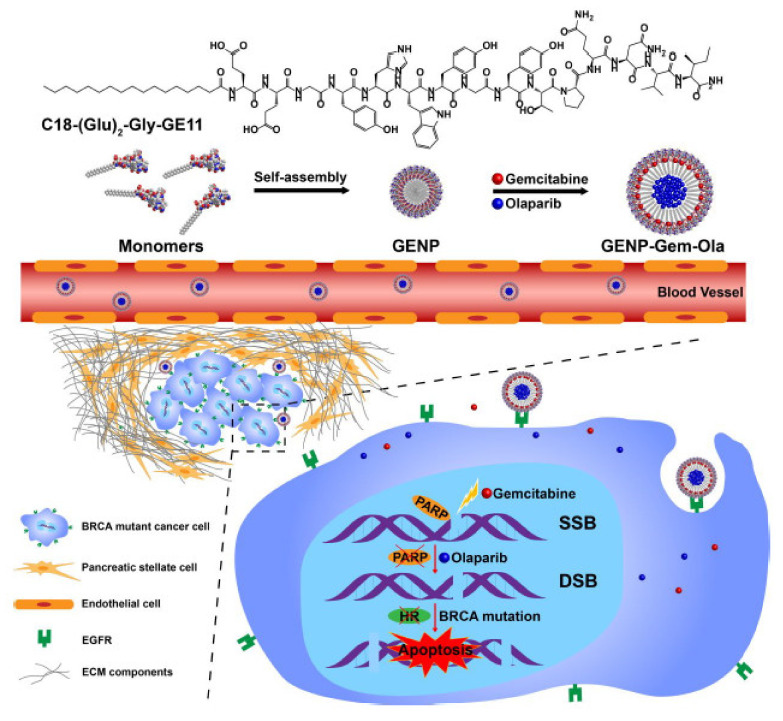
Self-assembly peptide nanoparticles (GENPs) that target EGFR and distribute gemcitabine and olaparib to pancreatic cancer cells. The mechanism that leads to cell apoptosis is represented by the inhibition of PARP through olaparib and DSBs generated by gemcitabine. Abbreviations: EGFR, epidermal growth factor receptor; ECM, extracellular matrix; PARP, poly ADP-ribose polymerase; DSBs, double-strand breaks. Reprinted with permission from [8].

Du et al. developed a self-assembly amphiphilic peptide nanoparticle (GENP) to co-deliver gemcitabine and the PARPi olaparib to target *BRCA* mutant gene in pancreatic cancer. The developed nanobiocompound was highly stable, showed a low cytotoxic effect on normal cells, was relatively easy to encapsulate, and equitably delivered the two therapeutic agents inside the tumor tissue (Figure 1).

The author demonstrated that gemcitabine and olaparib exhibited powerful in vitro reactions in optimal conditions. The nanoparticle extended the half-life of both chemotherapeutics and led to their tumor aggregation at the ideal therapeutic ratio in vivo.

The drug-charged nanoparticles were capable of considerably abolishing tumor expansion in a murine PCa model with minimal side effects. Drug co-distribution of DNA-altering agents and PARP blockers via the GENP expresses an anticipative way for the therapy of pancreatic malignancy [8].

A very difficult technique, that of squalene conjugation to gemcitabine and to form nanoparticles (NPs), proved to generate a very specific and sensitive therapeutic system against pancreatic cancer.

Squalenoylation conjugation chemistry was reinforced to enhance the therapeutic potential and adaptability using tert-butyldimethylsilyl (TBDMS) shielding groups. Tucci et al. then increased an adjustable microfluidic mingling program to create SqGem-based NPs and investigated the strength and morphology of the developed nanobiocompounds using dynamic light scattering (DLS) and transmission electron microscopy (TEM). Cytotoxicity was studied in both PANC-1 and KPC (KrasLSL-G12D/+; Trp53LSL-R172H/+; Pdx-Cre) pancreatic cancer cell lines. A 64Cu chelator (2-S-(4-aminobenzyl)-1,4,7-triazacyclononane-1,4,7-triacetic acid, NOTA) was squalenoylated and used with positron emission tomography (PET) imaging to study the in vivo therapeutic potential of SqGem-based NPs. 

The authors discovered that squalenoylation proceeds with increased gemcitabine absorption from 15% to 63%. Cholesterol-PEG-2k integration was used to develop SqGem-based NPs when employing this method, and the addition of extra cholesterol enhanced the stability of the developed nanobiocompound at room temperature; after 1 week, the PDI of SqGem NPs with cholesterol was ~0.2, whereas the PDI of SqGem NP deficiency cholesterol was ~0.5. Comparable or higher cytotoxicity was reached for SqGem-based NPs in comparison with gemcitabine or Abraxane® when the treatment was performed using a concentration of 10 µM. This method for the development of powerful squalenoylated gemcitabine nanoparticles holds great hopes for the future treatment of pancreatic cancer [9].

Gemcitabine is currently one of the most administered chemotherapeutic drugs in the therapy of pancreatic cancer. However, gemcitabine resistance is commonly met in many patients with pancreatic cancer. One of the biggest explanations is the weak hENT1 expression. Relying on the above hypothesis, Guo et al. explored the antitumor effect of gemcitabine loaded with human serum albumin nanoparticles (GEM-HSA-NPs) on gemcitabine-resistant pancreatic cancer induced by weak hENT1 expression. To induce the suppression of the hENT1 gene, S-(4-nitrobenzyl)-6-thioinosine was used to block its activity. Following the administration, cellular growth studies, cell cycle and apoptosis tests were performed on human pancreatic malignant cells BxPC-3 and SW1990. 

The in vivo antitumor result was investigated by using a patient-derived xenograft (PDX) pattern. The in vivo toxicity, pharmacokinetics and biodistribution were evaluated using adult Kunming mice. In in vitro experiments, GEM-HSA-NPs could block cell development, stop the cell cycle, and led to apoptosis when tumor cells were resistant to gemcitabine. In in vivo studies, GEM-HSA-NPs were more efficient than gemcitabine in suppressing tumor development in PDX models, regardless of the high or low hENT1 expression. The in vivo toxicity evaluation proved that the biotoxicity of GEM-HSA-NPs was not higher in comparison with gemcitabine. 

GEM-HSA-NPs can prevail over gemcitabine resistance due to minimal hENT1 levels, which is indicative of their promising function for pancreatic cancer treatment in humans [10].

## 8. Gemcitabine and Iron Oxide

Meaningful expression of the anti-phagocytosis signal CD47 has also been noticed in pancreatic cancer cells, mostly in cancer stem cells (CSCs), which are partly responsible for chemoresistance and for being aggressive. The CD47 receptor was shown on pancreatic cancer cells and clearly displayed on CSCs, but not on normal pancreas cells. Suppressing CD47 using monoclonal antibodies has been exploited recently to treat pancreatic cancer. In addition, CD47 reduction successfully lowered tumor proliferation only in association with gemcitabine or Abraxane. 

In this context, Trabulo et al. demonstrated the development of multifunctionalized iron oxide magnetic nanoparticles (MNPs) that incorporate the anti-CD47 antibody and the chemotherapeutic drug gemcitabine for the treatment of pancreatic cancer. They proved the in vitro effectiveness of the preparation following administration in CD47-positive pancreatic cancer cells [11].

## 9. Curcumin and Chitosan Np

In their work, Thakkar et al. investigated the chemopreventive efficiency of orally administered chitosan-coated solid–lipid nanoparticle (c-SLN) encapsulated aspirin (ASP), curcumin (CUR) and free sulforaphane (SFN), ACS-cSLN, in an LSL-Kras G12D/+; Pdx-1 Cre/+ transgenic mouse model of pancreatic ductal adenocarcinoma (PDAC). In vitro intracellular localization and the uptake of ODA-FITC-labeled ASP and CUR c-SLNs were examined in Panc-1 and MIA PaCa-2 cells by using fluorescence microscopy. LSL-Kras G12D/+; Pdx-1 Cre/+ transgenic mice (*n* = 30) were divided into five groups. Treatment groups were treated with ACS c-SLNs in three doses: low (2 + 4.5 + 0.16 mg/kg), medium (20 + 45 + 1.6 mg/kg) and high (60 + 135 + 4.8 mg/kg). After 20 weeks of treatment, mouse pancreases were collected, dyed and tallied, in accordance with different pancreatic intraepithelial neoplasm (PanIN) groups, by an uncontrolled observer.

In vitro, cellular feedback calculated on Panc-1 and MIA PaCa-2 cells culminated in greater fluorescence intensities, expressing the augmented cellular uptake of ASP and CUR c-SLNs. For more documentation, the inclusion of lysoID (red fluorescence) proved the position and uptake of ASP and CUR c-SLNs into the lysosome. In vivo therapy with ACS c-SLN for 20 weeks did not induce evident adverse effects on progress and no statistically significant dissimilarity in body weight was detected among the groups. Nevertheless, the weight (mean ± SEM) of the pancreas when the study finished was greater in the blank c-SLN group (223.6 ± 42.2 mg) related to low (138.0 ± 26.0 mg; not significant [NS]), medium (145.0 ± 9.0 mg; NS), and high (133.8 ± 20.3 mg; NS) ACS c-SLN-treated groups, proving the effectiveness of ACS c-SLN nanoformulations. The low, medium and high doses of the ACS c-SLN mixture showed a reduction in tumor frequency (PanIN count) by 16.6% (*p* < 0.01), 66.8% (*p* < 0.01), and 83.4% (*p* < 0.01), respectively. 

These reports provide more proof for applying a low, oral dose of a nanotechnology-based combined procedure for the chemoprevention of PDAC. [12]

Arya et al. investigated the top method to develop curcumin-loaded Poly d,l-lactide-co-glycolide (PLGA) NPs and also to cover the surface of it with chitosan and PEG (CNPs), expecting a decrease in restrictions linked with native curcumin distribution for obtaining the greatest therapeutic effect. In vitro, cellular investigations demonstrated better cytotoxicity and improved the therapeutic potential of CNPs in metastatic pancreatic cancer.

Therefore, the authors hope that the outcomes from these explorations can launch new possibilities for treating pancreatic cancer [13].

## 10. Silver Np

Zielinska et al. evaluated the capacity of AgNPs to suppress pancreatic cancer cells, and then determined the molecular process that led to this result. Therefore, Zielinska et al. estimated the cytotoxicity of AgNPs against non-cancerous cells of the same tissue (hTERT-HPNE cells). Their outcomes suggested that AgNPs with sizes of 2.6 and 18 nm reduced viability and growth and promoted extensive apoptosis in malignant pancreatic cancer cells in a volume–concentration relationship.

Examination of the cellular lines showed that the addition of AgNPs instigated the increased apoptosis of pancreatic cancer cells, the effect being linked to increasing the amount of Bax protein which has the ability to upregulate apoptosis, and decreasing the level of Bcl 2 protein, which downregulates apoptosis. Furthermore, AgNPs remarkably upregulated tumor repressor p53 protein levels. Additionally, they discovered that the pancreatic cell line was increasingly receptive to the toxicity generated by the silver nanoparticles. To conclude, AgNPs are able to cause various forms of automatic apoptosis in PANC-1 cells, which may possibly aid in the battle against chemoresistance [14]. Barcińska et al. presumed that the generation of oxygen toxicity by reactive oxygen species (ROS) or high, sustained levels of reactive nitrogen species (RNS) can lead to pancreatic cancer cells death. They chose to use silver nanoparticles (AgNPs) (2.6 and 18 nm) as a trigger for oxygen or nitrogen toxicity. The experiment was performed on a pancreatic cancer cell line, PANC-1. The authors had already established in a previous study that silver nanoparticles can lead to PANC-1 apoptosis. Additionally, it is acknowledged AgNPs can lead to ROS generation and accumulation in different types of tumors, and they are suggested to be a promising means for the development of oncologic treatments. Additionally, the purpose of their experiment was to determine the involvement of oxidative and nitro-oxidative stress in the cellular interactions between AgNPs and PANC-1 cells. They established that AgNPs can increase ROS levels in PANC-1 cells and pancreatic noncancer cells (hTERT-HPNE). They also discovered that the rise in ROS levels was weaker in non-cancerous cells. Depletion of mitochondrial membrane potential and modifications in the cell growth and division were noticed as well. Moreover, Barcińska et al. revealed that there was a significant generation of reactive nitrogen species, represented by nitric oxide and dioxide, by the PANC-1 cells, as well as interference with enzymes that have antioxidant proprieties, such as glutathione peroxidase or superoxide dismutase, in protein levels as well as microRNA levels. Moreover, the experiment revealed structural damage to the cells which was indicative of oxidative stress. They concluded that AgNPs have the ability to induce oxidative stress, which leads to apoptosis in pancreatic cancer cell lines [15].

## 11. Carbon Nanotubes

It is already a known fact that one of the deadliest malignancies in the world is pancreatic cancer. The current therapeutic options for advanced inoperable carcinomas are mostly inefficient; therefore, there is a vital need to search for a more effective anticancer approach. In the pursuit of new and better interventions, photothermal therapy (PTT) based on nanomaterials presents a growing interest. 

The latest progress in connected areas has motivated the generation of new nanoprobes, such as organic pigments and metal nanoparticles. However, these materials have shortcomings, for example: organic pigments have weak stability and are easily perishable, whereas metal nanoparticles are conceivably harmful to cells and hard to damage; both of them have low photothermal conversion effectiveness; there are a wide range of antineoplastic effects; and tumor targeting is inadequate. In this paper, the authors used single-wall carbon nanotubes (SWNTs) in order to overcome some of the barriers mentioned above. They discovered that SWNT proprieties, such as minimal cellular toxicity, permeability and stability, make them ideal for photothermal applications. Particular changes can ease the process of imaging the SWNTs at both macroscopic and microscopic levels in tumors. Photothermal therapy thus provides an encouraging new strategy in oncologic treatments, with high precision and fewer side effects.

Lu et al. demonstrated that, by using this approach, PTT provides accurate and exceptional therapeutic outcomes with reduced side effects; therefore, a promising approach in treating cancer has been achieved [16].

The biomarker CA 19-9 is the only one approved for its prognostic value in pancreatic cancer, but researchers have also tried to use it as a marker for targeted therapy, or in combination with nanotherapeutics, since it is highly specific to PC. Tsai et al [17] have measured CA 19-9 levels before and after PC surgery and neoadjuvant chemotherapy, proving that its levels before treatment can predict outcome, since failure to normalize was correlated with poor prognosis for this category of patients. However, another team of researchers point out that CA 19-9 levels can be elevated in diabetic patients, therefore proposing a cut-off of 75 U/mL for separating diabetics from pancreatic cancer patients [18]. This is important to keep in mind when evaluating this marker’s levels, especially as long as diabetes is a common disease. All things considered, the study strengthens the idea that CA 19-9 is a marker that should be thoroughly used and researched in PC.

To that end, Bhosale et al. described the assembly of a sensor used to detect the CA 19-9 biomarker. The team of researchers used electrospun nanofibers, which they linked with gold nanoparticles or carbon nanotubes in order to develop an immunosensor able to identify the biomarker through impedance spectroscopy. The immunosensors, which were either coupled with SWCNTs or gold nanoparticles, both demonstrated capability in detecting CA 19-9. The discrepancy between the two was represented by the lower limit of recognition with the gold nanoparticles.

The nanofibers used served as a template for restriction of the CA 19-9 antibodies; the antibody–antigen complex was irreversibly adsorbed, a process confirmed by spectroscopy imaging. Blood samples from patients were also used in order to detect the biomarker using the technology applied, with promising results. This detection process, based on immunosensors, could represent a new and improved detection method of early pancreatic cancer [19].

As stated, identifying the tumor marker carbohydrate antigen 19-9 (CA 19-9) in biological samples, within clinical practice, can enhance and promote the early detection of pancreatic cancer. 

A new, feasible method was proposed by a team of researchers in order to detect the biomarker CA 19-9 through the use of nanotechnology. By employing carbon quantum dots and gold nanocomposites with horseradish peroxidase as a labeling antibody agent, the team developed a fluorescence immunosensor assay that successfully detected the biomarker in serum samples. The boost in fluorescence was proportionate to the quantity of CA 19-9. The present study accomplished a highly sensitive detection limit, the lower limit being 0.007 U mL^−1^, which is by far the lowest compared to other techniques used. The authors point out that this is a simple method, reproducible, and eco-friendly. The composite used could therefore be a potential improved instrument in the detection of CA 19-9 [20].

## 12. Doxorubicin

**Figure 2:**
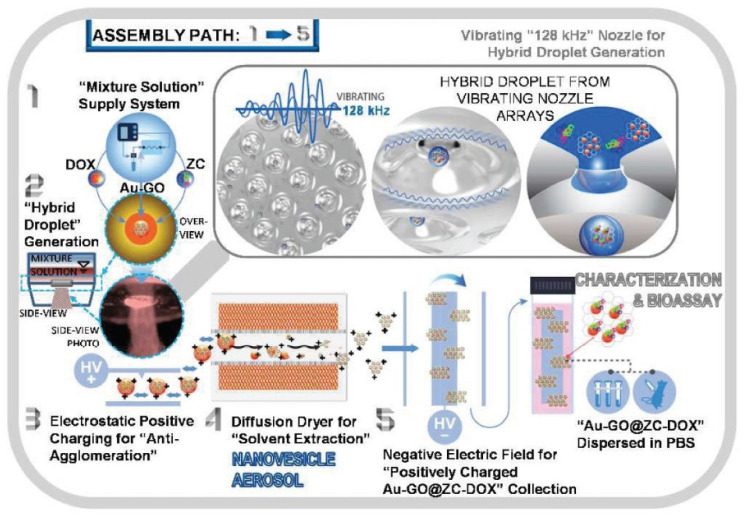
Assembly of the nanocomplex from gold–graphene, zwitterionic chitosan and doxorubicin, through a vibrating nozzle method. Step 1 is represented by the Au-GO being immersed in deionized water, together with ZC and DOX; step 2 is the connection of a vibrating nozzle to the bottle containing the complex to generate hybrid droplets; step 3—electrostatically charging the droplets; and step 4—generating the nanovesicles by passing the complex formed in step 3 through a diffusion dryer which detaches the solvent. Step 5—accumulating the nanovesicles on negative electric aluminum fields. Abbreviations: Au-GO, gold–graphene oxide; DOX, doxorubicin; ZC, zwitterionic chitosan. Reprinted with permission from [21].

New and continuous research in the development of improved drug delivery strategies has led to important findings in this field. The distribution of chemotherapeutics is impeded by various factors such as the tumor microenvironment, and it also involves great toxicity; therefore, researchers are trying to overcome these barriers by creating enhanced procedures to deliver drugs in pancreatic cancer. An interesting and novel technique was employed by a team of researchers in order to improve doxorubicin administration and photothermal therapy in pancreatic cancer cells (PANC-1 and MIA PaCa-2 lines). Researchers used gold nanoparticles and graphene oxide, which have photothermal capabilities and can absorb near-infrared waves; their combination has proved useful for photothermal applications (Figure 2). Zwitterionic chitosan (ZC) was used as a capping agent, to prevent the fast elimination of the nanocomposite. The nanoformulation used expressed excellent uptake, distribution, and antineoplastic effects. Additionally, it was minimally toxic. The approach may pave the way to improved therapy strategies for pancreatic cancer [21].

**Figure 3:**
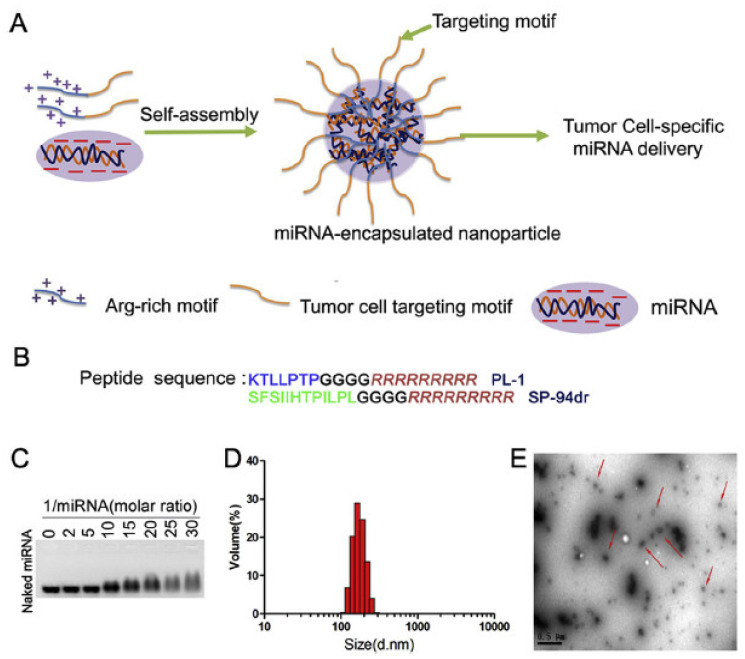
(**A**) The assembly of plectin-1 (PL-1) and miR-212 nanocomplex. (**B**) The PTP peptide is linked to the N-terminal of the arginine residues with a glycine linker. (**C**) An assay containing agarose gel was added to various PL-1 to miR-212 molar ratios. (**D**) The diameter of the nanoparticles was, on average, 180 ± 17 nm. (**E**) Transmission electron microscopy of the nanoparticles. Reprinted with permission from [22].

Pancreatic ductal adenocarcinoma (PDAC) a type of cancer with a poor prognosis. There is an urgent requirement for novel therapy objectives and approaches in order to reduce PDAC-related deaths. microRNA-212 (miR-212) was discovered to play a role in tumor suppression in many malignancies, although its latest role and definite structure in PDAC development is ambiguous. In the present study, Chen et al. developed a nanocomposite from plectin-1, which has the ability to target PDAC cells and RNA binding particles in order to deliver doxorubicin to PC cells. These nanoparticles can accurately convey miR-212 to pancreatic cancer cells, demonstrating favorable cohesion in RNase and serum as well. Additionally, the authors showed that the nanoparticles developed could completely augment the therapeutic efficacy of doxorubicin, in vivo and in vitro. The system is based on declining of USP9X expression (ubiquitin-specific peptidase 9, X-linked, USP9X) and finally intensifying the doxorubicin-induced apoptosis and autophagy of PDAC cells by combining miR-212 intervention with PL-1/miR-212 nanoparticles. The system was proven to amplify the chemotherapy effects and diminish the expression of the ubiquitin-specific peptidase 9 (Figure 3). These conclusions present a new, encouraging anti-cancer strategy through PL-1/miR-212 nanoparticles and suggest miR-212/USP9X as a possible instrument for a potential new therapeutic strategy in human PDAC [22].

## 13. Paclitaxel

In England, up to 69% of newly diagnosed pancreatic cancers are in metastatic stages. Apart from the known chemotherapeutics used in this stage, albumin-bound paclitaxel in combination with gemcitabine was recently approved as first-line treatment for stage IV PC, in Scotland and Wales, but not in England. To this point, the National Institute for Health and Care Excellence (NICE) Single Technology Appraisal (STA) process was applied in order to evaluate the cost-effectiveness and to identify the most suitable subgroup of cancer patients who would be eligible to receive albumin-bound paclitaxel and gemcitabine (Nab-Pac + Gem) for pancreatic cancer treatment. An independent review group was used for this purpose. The evidence presented was based on the CA046 trial, a phase III trial which compared the nanocomplex with gemcitabine alone, in the treatment of metastatic PC, which demonstrated improved survival, from 6.7 months in patients treated with gemcitabine to 8.5 months in the other group. In this analysis, Nab-Pac + Gem effectiveness was compared to standard gemcitabine (Gem), gemcitabine with capecitabine (Gem-Cap) or FORLFIRINOX (fluorouracil, irinotecan, oxaliplatin, leucovorin) treatment. The nanocomplex was proven to statistically improve survival only when compared to gemcitabine alone. No statistical difference was observed when compared to Gem-Cap or FORLFIRINOX. Additionally, cost-effectiveness studies did not show differences when comparing Nab-Pac+ Gem with Gem-Cap or FOLFIRINOX, although it was statistically significant compared with standard Gem. The main problem when discussing the implementation of another treatment in pancreatic cancer is represented by the lack of clinical guidelines for patient selection. There are no clear rules when it comes to choosing the best subpopulation that will benefit most from a certain type of treatment. Due to a lack of evidence, the group eventually concluded that patients should receive the nanocomplex only when the patients are not suitable to receive other types of treatments or would otherwise receive treatment with gemcitabine alone [23].

Pancreatic ductal adenocarcinoma (PDAC) is composed of an exaggerated desmoplastic stroma which has the ability to restrict the distribution of chemotherapy treatments within the tumoral cells and leads to radiotherapy resistance; therefore, stromal as well as tumor areas have to be targeted in the hopes of successfully curing PDAC. Zhao et al. therefore co-produced a sonic hedgehog suppressor, cyclopamine (CPA), and bound paclitaxel (PTX), a plant alkaloid with chemotherapeutic effects and a polymeric micelle (M-CPA/PTX). CPA can reduce the production of cancer-related fibroblasts (CAFs) by the stroma, whereas PTX is able to diminish cancer growth. In this study, Zhao et al. proved that in suitable PDAC models, M-CPA successfully affects stroma through enhancing the density of the microvasculature, diminishing the hypoxic medium, and lowering the rigidity of the extracellular matrix (ECM) while preserving the tumor-restraining viability of the ECM. M-CPA/PTX can considerably increase animal survival by inhibiting the proliferation of the cancer cells and diminishing the phenotypes which have the worse prognosis, namely, poorly or moderately differentiated. Their review recommends that utilizing functionalized nanoparticles that target both stromal and the tumor areas is an auspicious approach for curing PDAC [24].

## 14. Cetuximab

Photodynamic therapy (PDT) has revolutionized cancer treatment strategies since its implementation. Its basic principle is founded on the cells destroying themselves through reactive oxygen species, by using a photosensitizer (PS) drug and light. However, not all drugs that act as PSs have good photophysical characteristics, and this is where nanotechnology, once again, proves its worth. The numerous advantages as drug carriers has caused its use in the PDT domain to rise significantly. Mesoporous silica nanoparticles (MSNPs) are easy to develop, have a sizeable surface area, are biocompatible, and can easily be functionalized with different ligands; all these advantages have made them of great interest for use in PDT. To this end, a team of researchers developed a nanocomplex from MSNPs with cetuximab (an epidermal growth factor receptor targeting antibody) and used it on pancreatic cancer cell line to explore its potential use for PDT, as well as cellular uptake and ROS production. The cellular lines used were represented by PANC-1, MIA PaCa2 and AsPC-1. In vivo and in vitro studies showed the greatest uptake and phototoxic effects for the nanocomplex conjugated with cetuximab, because most pancreatic cancer cells express EGFR on their surface. The novel system demonstrated excellent results and could be implemented as a new therapeutic strategy for the selective delivery of chemotherapy and PDT in pancreatic cancer [25].

## 15. Gene Therapy

Genetics and genes have a massive influence on all types of cancers, including pancreatic cancer. Numerous studies have demonstrated the involvement of genes such as *KRAS*, or cycle regulators such as p53 or p16 in pancreatic cancer. When activated, *KRAS* stimulates cell proliferation, especially in the early stages; regarding p53 or p16, studies have demonstrated that they are inactivated in most PCs. It is therefore reasonable that research should focus on developing gene therapies for this type of cancer. Nanoparticles have proved useful as drug carriers, Kurtanich et al. focused on gene therapy using nanoparticles and other recent developments. Their team tackled the subject of gene augmentation, where a defective gene is replaced with a normal one; for example, EGFR gelatin nanoparticles were used to deliver *p53* genes to PC cancer cells; the nanocomplex was proven to augment the apoptosis of the cancer cells; to the same effect, gelatin nanoparticles with gemcitabine were used to deliver *p53* genes, which resulted in a 77.3% tumor growth inhibition. microRNA therapies were also used in combination with nanotechno-logy for effective drug delivery and tumor inhibition. All these studies prove that gene delivery represents a potential new therapeutic strategy, and although most of them were only tested in vitro, their immense potential needs to be further explored in order to properly implement this type of treatment [26].

## 16. K-ras Gene Mutations 

Regarding the detection of mutated genes, specifically *K-ras* mutation, which, as stated, is related to disease progression in PC, a team of researchers proved that nanotechnology can be useful in this domain as well. *K-ras* mutation is an early event, and various studies have identified its presence in biopsy specimens or different biological samples from pancreatic cancer patients; however, this team developed a new strategy for its detection, from fecal samples of patients with different benign or malign pancreatic diseases. DNA was extracted from the fecal samples, and *K-ras* mutation was detected using magnetic nanoparticles as a nanoprobe. The mutation rate of *K-ras* in advanced pancreatic cancer was high—84.0%. The specificity and sensitivity of the detected mutation were 81.8% and 81.5%, respectively, although when the method was combined with CA 19-9 detection, the sensitivity was 97.7% and the specificity was 80.9%. When simply compared to CA 19-9, *K-ras* mutation’s sensitivity and specificity were higher, but not statistically significant. The authors also pointed out that the mutation rate was almost equal in patients with early pancreatic cancer, compared to advanced PC, augmenting the idea that *K-ras* mutation appears early in the development of this type of cancer. The study proves that *K-ras* mutation could be detected from fecal samples in high-risk populations using nanoparticles, and that combining this method with CA 19-9 detection further amplifies its advantages [27]. The method needs to be tested on greater cohorts before its clinical implementation, but because it is a non-invasive, fairly feasible detection technique, it seems reasonable to apply it. 

## 17. Retinoic Acid-Inducible Gene I

Pancreatic cancer is associated with poor chemotherapy and immunotherapy responses. Effective drug delivery is impeded by the structure of the tumor microenvironment and its immunosuppressive characteristics. Researchers have tried to develop new methods for modulating the TME while increasing tumor cell apoptosis, and nanotechnology has been of great help in this area, too. Das et al. used lipid calcium phosphate nanoparticles (LCP-NPs) which encapsuled double-stranded RNA (dsRNA) on an orthotopic KPC model of PDAC. dsRNA is an agonist for retinoic acid-inducible gene *I* (*RIG-I*)-like receptors and the dsRNA interaction with RIG-1-like receptors led to a cascade of cellular processes that eventually finished with the induction of pro-inflammatory cells, such as type I interferon or Th1 cytokines, and a decrease in B cells, consequently remodeling the immunosuppressive TME. dsRNA also silences *Bcl2*, a process that led to augmented cell apoptosis. The nanocomplex thus increased survival and significantly inhibited tumor growth, providing a new immunomodulatory treatment strategy [28].

## 18. Ectopic High Endothelial Venules

Nanomedicine provides a notable chance to undertake treatment-refractory malignancies by increasing the distribution of drugs to the tumor site. A team of researchers tried to identify high endothelial venules (HEVs) as targets for therapy; they are usually found in lymph nodes or can appear in inflammatory processes. They express a molecule which is recognized by MECA79 monoclonal antibody; using this knowledge, the authors developed this to identify ectopic HEVs in pancreatic cancer. The accumulation of the conjugated nanoparticles to the tumor site was greater compared to non-conjugated nanoparticles. They further added paclitaxel (Taxol) to the nanoconjugate; the nanocomplex managed to improve drug delivery to the malignant site and substantially decrease tumor volume. In addition to this result, the nanocomplex led to the apoptosis of a higher proportion of cancer cells, as well as to a decrease in vascularization in the tumor site. The study proved that HEVs may represent new therapeutic targets in the fight against pancreatic cancer [2].

## 19. Np-Based Micelles

Han et al. formulated an easy approach for elaborating upconversion nanoparticles (UCNPs) with gadolinium and the epithelial adhesion molecule CD326 and conducted the matching specifications. After applying cellular tests, they further studied the dynamic targeting ability of the micelles in dual mode imaging. The authors discovered that the nanocomplex possessed better imaging characteristics and longstanding permanence. By carrying out in vitro and in vivo experiments, the authors concluded that the nanoformulation had excellence biocompatibility. They identified further micelle uptake of the tumoral cells facilitated by the CD326 molecule attached onto the micelles. Additionally, Han et al. efficaciously determined that CD326-conjugated nanoparticles conferred targeting capacity by augmenting the dual imaging performance in orthotopic models of pancreatic cancer. Along with great biocompatibility and high imaging attributes, the nanoformulation proved to possess a high capacity for detecting early pancreatic cancer in the future and suggests a role in facilitating the next biomedical submission [29].

## 20. Salinomycin

Salinomycin (SAL), an antibiotic, was recently discovered to have a potential role in inhibiting multi-drug-resistant cancer cells and to inhibit cancer cell proliferation. Daman et al. used poly (lactic-co-glycolic acid) (PLGA) nanoparticles loaded with SAL on an orthotopic model of PC in order to evaluate the potential therapeutic benefit of SALs. After in vitro assessment of the cytotoxicity of the nanoparticles, they were inserted in the pancreas of nude mice. After three weeks of therapy, administering 3.5 mg/kg once every two days, a reduction in tumor size up to 52% was obtained. Western blot analysis revealed that SAL can lead to high levels of E-cadherin or β-catenin, thus harnessing the extracellular matrix. Its utilization also increased apoptosis of the tumoral cells, but not in the stroma, as revealed by immunofluorescence studies. Daman et al. concluded that the nanocomplex used could represent a potential method for pancreatic cancer therapy, although the engineering considerations should be additionally explored [30].

## 21. Hydrogen Peroxide

Recently, synergistic approaches to cancer treatment have gained a lot of ground, because combining photodynamic therapy (PDT) with photothermal therapy (PTT) has led to very promising results. To this end, Wang et al. developed a nanoformulation composed of iron oxide nanoparticles integrated with indocyanine green (ICG) as a photosensitizer and hyaluronic acid on the surface, to be used for synergistic PDT and PTT treatment of pancreatic cancer cells. The complex triggered the Fenton reaction intracellularly, which subsequently led to the release of reactive oxygen species. The platform revealed good results, with increased biocompatibility and good guiding capacity. The team also discovered that the surplus of hydrogen peroxide can be used to provide ROS that can kill cells through Fenton reactions. In conclusion, the nanocomplex developed achieved effective phototherapy results, providing a potential new therapeutic and diagnostic instrument [31].

## 22. Metformin

Guiding glutamine metabolism through pharmacological suppression of glutaminase has been converted into many clinical tests as a new cancer treatment. In this report, Elgogary et al. used an exclusive solubilization technique to encapsulate bis-2-(5-phenylacetamido-1,2,4-thiadiazol-2-yl) ethyl sulfide (BPTES), a rather insoluble glutaminase suppressor, in nanoparticles. The authors used this particular method because tumor cells which are fast-dividing utilize glutamine, and the analysis of different pancreatic cell lines revealed that they are glutamine-dependent in regard to proliferation; therefore, administering a nanoparticle that contains a suppressor of the glutamine metabolism will lead to apoptosis. The nanocomplex showed enhanced pharmacokinetics and efficiency in comparison with unencapsulated BPTES. Furthermore, tumor cells that are hypoxic metabolize glucose and are an effective target for an antidiabetic drug such as metformin. The combination of the nanocomplex with metformin administration led to significant tumor growth inhibition in pancreatic cancer cells, more than when being administered alone [32]. Researchers have also discovered that the inhibition of glutaminase only leads to the suppression of growing cancer cells, not the hypoxic cells. Taking into consideration these results, they concluded that targeting more than one metabolic direction could be a new and efficient treatment strategy in pancreatic cancer. 

## 23. iRGD Peptide 

Many solid tumor forms, such as pancreatic cancer, mostly have a low prognosis, partly considering that the distribution of therapeutics is restricted by pathological deviations that intercept connections with tumor vascularization, thus leading to poor drug distribution. In trying to overcome this barrier, researchers have developed a nanocomplex containing a peptide that has tumor-penetrating characteristics, namely, iRGD. This peptide is efficient in promoting drug penetration into the tumor cells through transcytosis; therefore, a leaky vasculature is not necessitated. There have been many successful reports of different types of nanoparticles, such as liposomes, exosomes or inorganic nanoparticles that incorporate iRGD in solid tumors. Regarding pancreatic cancer, lipid micelles or protein nanocages with iRGD lead to effective drug delivery and tumor cell inhibition. In *K-ras*-mutated orthotopic models, the conjugation effect of nanocarriers loaded with iRGD was similar to other tumor types. All this research proves that this type of peptide can represent a promising tool for improving the chemotherapy response in pancreatic cancer [33].

## 24. Plectin-1 

Biomarkers as targets for pancreatic cancer therapy and imaging contribute to encouraging new therapeutic strategies. Further conjugating them with nanoparticles improves tumor detection, targeting, and increases the drug effect. Plectin-1 is a protein that is overexpressed in pancreatic cancer—one study discovered its expression in 93% of pancreatic adenocarcinomas; therefore, researchers tried to use it in combination with magnetic nanoparticles on four different pancreatic cancer cell lines and an orthotopic model, to examine their accumulation through various imaging techniques. To this end, they used superparamagnetic iron oxide nanoparticles (SPIONs), which they conjugated with plectin-1 antibody and administered it. Imaging methods for the detection of pancreatic cancer, such as CT or magnetic resonance imaging (MRI), do not detect this type of tumor in its early stage; recent research has tried to use nanoparticles in order to improve imaging data for this category of patients. The nanocomplex administered was internalized by the pancreatic tumor cells in vitro and in vivo; this was demonstrated through optical imaging and MRI. Therefore, a dual-functioning nanoprobe proved preferential accumulation and improved imaging, once again emphasizing the idea that combining treatment strategies could one day lead to significant improvements in PC treatment [34].

## 25. Arginine–Glycine–Aspartic Acid Peptide

The developments in recent research regarding nanomaterials led to the discovery of quantum dots (QDs), which have been extensively used in experiments; their conjugation with different molecules can lead to unique optical proprieties of the nanocomplex. Arginine–glycine–aspartic acid (RGD) is a peptide that can link to a cell adhesion integrin which is only expressed in cancerous cells. Using this knowledge, Ming-Li et al. developed a nanoprobe: RGD-conjugated nanoprobes, which have the potential to act as photosensitizers for photodynamic therapy. They administered the probe to tumor-bearing mice and acquired in vivo imaging of the nanoprobe. They also performed histological analysis. The nanoprobes were administered intravenously and intratumorally, with the latter having achieved the highest fluorescence, meaning that administering the nanoparticles directly into the tumor cells was significantly more effective for nanoparticle accumulation. Histopathologic examination of the major organs such as the liver or the kidney showed minimal toxicity. The experiment proved that RGD-conjugated QDs are good candidates for imaging and therapeutic applications, possessing various additional advantages such as low cost, easy preparation and low toxicity [35]. 

## 26. Fluorescence Nanobiosensors for Ultrasensitive (Sub-Femtomolar) Arginase and Protease Identification 

Little progress has been made in the field of the early detection of pancreatic cancer, although liquid biopsies are fast emerging techniques that could be of great help for early diagnosis in different types of cancers. Proteases with a tumor-promoting role, such as matrix metalloproteinases (MMPs), cysteine proteases, and arginase, have all demonstrated a substantial role in promoting pancreatic tumor cell progression. A team of researchers developed a fluorescence nanobiosensor for the detection of different biomarkers from liquid biopsies of patients with pancreatic neoplasia as well as healthy controls. They established that protease expression was significantly higher in pancreatic cancer lines compared to control groups. Their experiment revealed that cathepsin B, MMP-1 and MMP-2, arginase and urokinase plasminogen activators, detected through liquid biopsy, represent great biomarkers for the detection of PC, thus establishing an enzymatic signature for PC and also proving that nanoparticles can be of use in the early detection of this type of cancer [36,37,38,39].

## 27. Conclusions

Forthcoming efforts ought to focus on improvements in early detection approaches of pancreatic carcinoma with the aim of lowering the mortality rate. Novel therapies have been developed to overcome all these therapeutic issues, and the development of active nanocarriers represents an exciting opportunity to improve PC outcome. (Table 1). Additionally, there is a clear need for a more updated pursuit of therapeutic means with simple ways to test them in a clinical setting, in order to be authorized and implemented. Plenty of pharmaceuticals that are widely known to fight pancreatic cancer have not so far been included in nanocarrier systems. Further investigations among these anti-tumor agents may clarify the issues of resilience and provide fresh prospects for the disease. 

The using of nanocarriers in cancer therapy is very promising for further study. Nevertheless, research in the field of nano-toxicology requires greater attention. There are still some fields such as long-term toxicity studies that have not been performed as of yet (for example, kidney nanoparticle buildup), and obviously, improvements will be made regarding “Theranostic nano-medicine” by expanding novel versatile nanoparticles linking the skills of detection and the potential of therapy of pancreatic cancer, together with checking the medication delivery and its dispensation [35].

In conclusion, the latest pancreatic cancer therapies rely on nanotechnology systems and manage to improve the healing efficiency and enable the guiding of cancer cells. Additionally, it provides the patients with the advantages of small particle dimensions which strengthen their infiltration abilities and medical efficacy.

Moreover, combining nanocarriers into a bioavailable matter presents a considerable development in security and targeting alternatives.

Another interesting focus that has motivated research scientists due to its increased harmlessness is herbal-based therapy conjugated with nanocarriers. Similarly, detection and diagnostic methods have been elaborated considerably by utilizing nanotechnology, enabling untimely and powerful identifications of pancreatic malignancy.

In summary, even though extra research is required, the recent advancements in targeting PCSCs are expected to have strong impacts on therapy for PDAC in the future.

Accordingly, improved biological and pathophysiological understandings of PCSCs, connected with the progress of nanoparticle engineering, is expected to be vital for the establishment of novel valid therapies of pancreatic cancer [36].

## Figures and Tables

**Table 1:** Nanoparticles used for pancreatic cancer treatment.

Nr. Crt.	Author	Type of Nanoparticle	Size/Characteristics	Pathology	Mechanism of Action
**1.**	[31]	Hydrogen peroxide responsive iron oxide multifunctional nanoplatform (IONPs-ICG-HA)	~50 nm	Pancreatic cancer	Via intracellular Fenton reaction, synergistic photodynamic therapy and photothermal therapy
**2.**	[33]	iRGD-mediated tumor-targeted Np; tumor-penetrating iRGD peptides (CRGDĳK/R]GPĳD/E]C)	<0.5 nm	Pancreatic cancer	ranscytosis mechanism Phage screening method
**3.**	[22]	microRNA-212 (miR-212)	Hydrodynamic diameters	Pancreatic ductal adenocarcinoma (PDAC)	Combined miR-212 intervention by PL-1/miR-212 nanoparticles (PL-1 and SP-94R were synthesized by solid-phase synthesis and purified by high-performance liquid chromatography)
**4.**	[28]	LCP-AEAA NPs encapsulating ppp dsRNA, a RIG-I agonist and Bcl2 gene silencing agent	Around 32 nm with a subsequent higher peak at 255 nm	Pancreatic ductal adenocarcinoma (PDAC)	Gene silencing
**5.**	[19]	Nanostructured mats of electrospun nanofibers of polyamide 6 and poly(allylamine hydrochloride) coated either with multiwalled carbon nanotubes (MWCNTs) or gold nanoparticles (AuNPs), suitable for the immobilization of anti-CA19-9 antibodies to detect the pancreatic cancer biomarker CA19-9	Frequency range between 1 and 106 Hz with a 10 mV amplitude	Pancreatic cancer	Detection of CA19-9 is performed here with electrochemical impedance spectroscopy Citrate-functionalized AuNPs were synthesized using the Turkevich method
**6.**	[8]	Epidermal growth factor receptor (EGFR) targeting (with GE11 peptide) self-assembly amphiphilic peptide nanoparticle (GENP) to co-deliver gemcitabine and the PARPi olaparib	~30 nm (GENP) ~60–120 nm(GENP-Gem-Ola)	BRCA2 mutant pancreatic cancer	Immunohistochemistry High-performance liquid chromatography (HPLC) and electrospray ionization mass spectrometry
**7.**	[4]	ORI-GPC1-NPs: theranostic nanoparticle (NP) based on gold nanocages (AuNCs) modified with hyaluronic acid (HA) and conjugated with anti-Glypican-1 (anti-GPC1) antibody, oridonin (ORI), gadolinium (Gd), and Cy7 dye	~243 nm	Pancreatic ductal adenocarcinoma (PDAC)	Electro static adsorption method
**8.**	[13]	Curcumin-loaded Poly D,L-lactide-co-glycolide (PLGA) NPs and further surface-coated with chitosan and PEG (CNPs)	~264 nm	Pancreatic adenocarcinoma	FTIR analysis Annexin V assay by flow cytometer
**9.**	[21]	Hybrid droplets containing gold-graphene oxide (Au-GO), doxorubicin (DOX), and zwitterionic chitosan (ZC) for the assembly of Au-GO@ZC-DOX stealth nanovesicles (NVs)	~53.0 nm	Pancreatic cancer	A single-pass diffusion drying process without any hydrothermal reactions, separations, or purifications
**10.**	[32]	bis-2-(5-phenylacetamido-1,2,4-thiadiazol-2-yl)ethyl sulfide (BPTES)	sub-100 nm nanoparticles	Pancreatic ductal adenocarcinoma (PDAC)	A unique emulsification process, which uses sucrose esters as nanoemulsion stabilizers to achieve high PEG density on the nanoparticle surface
